# Psychobiotics Regulate the Anxiety Symptoms in Carriers of Allele A of IL-1*β* Gene: A Randomized, Placebo-Controlled Clinical Trial

**DOI:** 10.1155/2020/2346126

**Published:** 2020-01-07

**Authors:** P. Gualtieri, M. Marchetti, G. Cioccoloni, A. De Lorenzo, L. Romano, A. Cammarano, C. Colica, R. Condò, L. Di Renzo

**Affiliations:** ^1^Section of Clinical Nutrition and Nutrigenomic, Department of Biomedicine and Prevention, University of Rome Tor Vergata, Via Montpellier 1, 00133 Rome, Italy; ^2^School of Specialization in Food Sciences, University of Rome Tor Vergata, Rome, Italy; ^3^PhD School of Applied Medical-Surgical Sciences, University of Rome Tor Vergata, Via Montpellier 1, 00133 Rome, Italy; ^4^Casa di Cura Madonna dello Scoglio S.r.l. e SADEL di Salvatore Baffa S.p.a., Cotronei (KR), Italy; ^5^Department of Biomedicine and Prevention, University of Rome Tor Vergata, Via Montpellier 1, 00133 Rome, Italy; ^6^CNR, IBFM UOS of Germaneto, University “Magna Graecia” of Catanzaro, Campus “Salvatore Venuta”, 88100, Germaneto, Catanzaro, Italy; ^7^Department of Clinical Sciences and Translational Medicine, University of Rome Tor Vergata, Via Montpellier 1, 00133 Rome, Italy

## Abstract

**Background:**

Probiotic oral intake, via modulation of the microbiota-gut-brain axis, can impact brain activity, mood, and behavior; therefore, it may be beneficial against psychological distress and anxiety disorders. Inflammatory cytokines can influence the onset and progression of several neurodegenerative mood disorders, and the IL-1*β* rs16944 SNP is related to high cytokine levels and potentially affects mood disorders. The aim of this study was to examine the combined effect of IL-1*β* polymorphism and probiotic administration in mood disorder phenotypes in the Italian population.

**Methods:**

150 subjects were randomized into two different groups, probiotic oral suspension group (POSG) and placebo control group (PCG), and received the relative treatment for 12 weeks. Psychological profile assessment by Hamilton Anxiety Rating Scale (HAM-A), Body Uneasiness Test (BUT), and Symptom Checklist 90-Revised (SCL90R) was administered to all volunteers. Genotyping was performed on DNA extracted from salivary samples.

**Results:**

After 12 weeks of intervention, a significant reduction of HAM-A total score was detected in the POSG (*p* < 0.01), compared to the PCG. Furthermore, IL-1*β* carriers have moderate risk to develop anxiety (OR = 5.90), and in POSG IL-1*β* carriers, we observed a reduction of HAM-A score (*p* = 0.02).

**Conclusions:**

Consumption of probiotics mitigates anxiety symptoms, especially in healthy adults with the minor A allele of rs16944 as a risk factor. Our results encourage the use of probiotics in anxiety disorders and suggest genetic association studies for psychobiotic-personalized therapy.

## 1. Introduction

In the last years, the increased scientific interest about microbiota and its relationship with health maintenance and disease onset underlined the importance of bacterial composition in the gastrointestinal tract. In the neuroscience field, the recognized complex bidirectional communication between host microbiota and brain-gut axis opened to new discoveries on the neurological disorders and disease onset and tailored treatments for affected patients.

Two neuroanatomical pathways are involved in the brain-gut interaction. The central nervous system (CNS) shares information with the lumen and the enteric nervous system (ENS) [1], through the sympathetic and parasympathetic branches of the autonomic nervous system (ANS), and they mutually modulate gut functions and environment [2, 3].

Secondly, psychophysical stress can set off adaptive processes by the neuroendocrine system, which in turn regulates the hypothalamic-pituitary-adrenal (HPA) axis, increasing the levels of inflammatory cytokines and prostaglandins in the gut. These events lead to changes in the microbiota composition and increased gastrointestinal permeability [4].

HPA axis activity influences physiological and behavioral states, including anxiety and depressive disorders [5]. A balanced microbiota-gut-brain (MGB) axis improves CNS and ENS functions [4–6]. Conversely, MGB axis impairment damages gut microbiota, destroys intestinal epithelium integrity, and affects permeability, increasing circulating levels of endotoxin, bacterial lipopolysaccharide (LPS), and inflammatory mediators [7]. Currently, it is well known that intestinal inflammation and gut microbiota imbalance are related to chronic abdominal pain syndromes and eating disorders, and increasing evidences highlighted a link between gut microbiota and neurological and psychiatric disorders, such as anxiety and depression [8, 9].

Anxiety is a feeling characterized by agitation, anguish, fear, and disproportionate worry, usually without triggers, accompanied by various somatic signs [10]. This disturbance is related to different adverse health outcomes, especially in the elderly [11].

*In vivo* studies observed the development of anxiety signs and symptoms after fecal microbiota transplants, highlighting the ability to affect neuropsychiatric conditions through the changes of the microbial composition [12]. Although these effects were observed also in germ-free mice [13], this suggests to use probiotics as a treatment in neuropsychiatric disorders.

For this purpose, *Lactobacillus*, *Bifidobacterium*, and others species were used in animal and human studies as probiotic supplements to enhance the biodiversity and health of the gut microbiota [14] and to treat anxiety disorders, through the improvement of the MGB axis balance [15], obtaining the title “psychobiotics.”

Interleukin-1 beta (IL-1*β*) was clearly identified as an important player in the onset and progression of several neurodegenerative diseases [16], and numerous studies have already proved the link between mood disorder symptoms and proinflammatory cytokine expression and circulating levels [17].

Psychophysical stress increases the proinflammatory cytokines through the HPA axis, impairing the gut barrier integrity and causing dysbiosis related to anxiety disorder [18–21]. In particular, IL-1*β*, after a psychophysiological stress stimulus, can affect the gut microbiota balance and mood status [22]. The IL-1*β* expression is strongly influenced by some polymorphisms in the IL-1*β* gene, which increase the related cytokine levels, thus affecting the magnitude of inflammatory disorders, making them a determining cofactor in several chronic diseases and potentially in the onset mood disorders [23].

In particular, increased levels of IL-1*β* were observed in the presence of the rs16944 polymorphism (NM_0000576.2:c.-598T>C), which is found in the promoter region of IL-1*β* [24]. The rs16944 is located in the functional promoter region (https://www.ncbi.nlm.nih.gov/variation/tools/1000genomes/). The presence of the allele A of rs16944 increases the IL-1*β* production, and it was associated with elevated risk of depression in schizophrenic spectrum disorders [25], depressive symptoms in Alzheimer disease [26, 27], and depressed state in breast cancer patients [28]. The relationship between IL-1*β* polymorphism and anxiety disorder was observed by Kovacs et al. [29], but no other study has investigated the combined effect of IL-1*β* polymorphism and probiotic administration in mood disorder phenotypes.

Therefore, in the present study, we investigated if the administration of psychobiotic suspension could represent a novel, safe, and long-term solution to treat or prevent anxiety disorders, to reduce associated symptoms, and to ameliorate their psychological state, in carriers of IL-1*β* rs16944 gene polymorphism. To this end, a randomized, placebo-controlled clinical trial was conducted on female and male volunteers.

In this study, the primary objective was to investigate the effects of the SNP rs16944 within the IL-1*β* gene on anxiety development in a sample of the Italian population. The secondary outcome was to evaluate the possible beneficial effect of a new probiotic formulation on anxiety and related symptoms according to the IL-1*β* SNP rs16944. The third objective was to assess a change in body shape perception before and after probiotic intervention. To this end, a randomized, placebo-controlled clinical trial was conducted on volunteers.

## 2. Materials and Methods

### 2.1. Study Design and Outcomes

The study protocol was conducted between January 2017 and July 2017, using an interventional randomized placebo-controlled clinical trial. At the time of recruitment, the patients were submitted to nutritional status and psychometric test evaluation. A medical history was performed and saliva samples were collected. The subjects received the psychobiotic mixture or the placebo at the beginning of the trial with consumption instructions. Volunteers consumed the relative treatments at home, once daily (1 sachet/day), two hours before lunch, in order to ensure adequate gastrointestinal transit and absorption. Eligible patients were randomly divided into two groups: (1) psychobiotic oral suspension group (POSG) and (2) placebo control group (PCG).

Both groups followed the assigned treatment for a 12-week period. The subjects were asked to maintain their usual lifestyle and dietary habits and to report any illness or adverse reaction emerging during study conduction. The subjects were asked to report any missed consumption of the products during the intervention. The POSG and PCG arms were double-blinded. The subjects repeated nutritional visit 12 weeks after intervention initiation of each arm (±3 days).

Nutritional status evaluation, psychometric tests, and buccal mucosa sample extraction were carried out at the time of enrollment (T0) and after the 12-week period intervention (T1). All participants recruited into the study authorized their participation by reading and signing the informed consent form, drafted in accordance to the provisions of the Ethics Committee of Medicine, University of Rome “Tor Vergata,” and with the Helsinki Declaration of 1975, as revised in 1983. Trial registration: this protocol has been registered with ClinicalTrials.gov Id: NCT01890070.

### 2.2. Subjects

150 volunteers were initially recruited during routine medical check-up visits at the Section of Biomedicine and Prevention, Division of Clinical Nutrition and Nutrigenomics of the University of Rome “Tor Vergata.” Exclusion criteria were age < 18 and >65, pregnant and lactating women, type 1 diabetes, established altered intestinal bacterial flora (intestinal bacterial overgrowth), history of psychiatric or psychological disturbance, absence of depression evaluated with Symptom Checklist-90 (SCL90) Global Severity Index (GSI) (score < 1), acute disease, endocrine, metabolic, liver, and gastrointestinal disease, cardiovascular or kidney dysfunction, cancer, and HIV infection. Subjects that were recently under antibiotic treatments, chronic pharmacological therapy with anti-inflammatory drugs or oral contraceptives, other probiotics or dietary supplements, subjects who are following dietary treatments, smokers, and alcohol and drug abusers were also excluded from the protocol. No subjects with known alterations of intestinal transit following organic pathologies (abdominal surgery, diabetes mellitus, scleroderma, hypothyroidism, etc.) were included in the study. The subjects enrolled into the study were asked to not consume any other probiotics or food supplements for the whole duration of the study.

### 2.3. Interleukin 1 Beta Genotyping

The DNA extraction from salivary samples collected with swabs was performed according to Hochmeister et al. [30]. gDNA was quantified with NanoDrop. Master Mix Taq DNA Polymerase and dNTPs (TaqPath ProAmp Master Mix, Life Technologies, CA, USA) and a two allele-specific fluorescent probes (TaqMan SNP Genotyping Assays, Life Technologies, CA, USA) were used to prepare the gDNA for the genotyping. The IL-1*β* gene rs16944 (NM_0000576.2:c.-598T>C) context sequence was as follows: TACCTTGGGTGCTGTTCTCTGCCTC(G/A)GGAGCTCTCTGTCAATTGCAGGAGC.

Genotyping was carried out using the StepOnePlus™ Real-Time PCR System (Applied Biosystems StepOnePlus Real-Time PCR, Life Technologies, CA, USA), according to the manufacturer's instructions.

### 2.4. Psychodiagnostic Instruments

#### 2.4.1. Hamilton Anxiety Rating Scale (HAM-A)

The Hamilton Anxiety Rating Scale (HAM-A) revised version questionnaire consists of 14 items used to define several anxiety-related symptoms, including both psychological and somatic symptomatology. The 14 items included are as follows: anxious mood; tension (startles, restlessness, and crying); fears (dark/strangers/crowds/animals); insomnia; “intellectual” (poor memory/difficulty concentrating); depressed mood (including anhedonia); somatic symptoms (aches, stiffness, and bruxism); sensory (tinnitus, blurred vision); cardiovascular (e.g., tachycardia and palpitations); respiratory (chest tightness, choking); gastrointestinal (irritable bowel syndrome-type symptoms); genitourinary (urinary frequency, impotence); autonomic (dry mouth, tension headache), and observed behavior at interview (restless, fidgety, etc.) [27].

In this study, HAM-A was administered by instructed physicians pre- and posttreatment. To each item, a score between 0 and 4 was attributed, considering 0 the absence and 4 the presence of severe symptoms. The total score ranges from 0 to 56 and was interpreted as follows: <17 mild anxiety, 17-24 mild-moderate anxiety, and 25-30 moderate-high anxiety.

Anxious individuals were considered the ones that had a score equal to or higher than 18 (≥18).

#### 2.4.2. Body Uneasiness Test (BUT)

The Body Uneasiness Test (BUT) is a self-assessment scale used for body image studies and related pathologies. BUT allows to calculate the Global Severity Index (GSI) or total average score, which is obtained from the sum of clinical scores (BUT-A), divided by their number (34). Item number with score ≥ 1 corresponds to Positive Symptom Total (PST). The sum of item scores ≥ 1 divided by PST produces the Positive Symptom Distress Index (PSDI) [31].

Five factors were defined: WP (Weight Phobia), BIC (Body Image Concerns), A (Avoidance), CSM (Compulsive Self-Monitoring), and D (Depersonalization). In our study, we considered as positive for altered perception of body image a GSI score ≥ 1.2.

#### 2.4.3. Symptom Checklist-Revised (SCL90R)

Symptom Checklist-Revised (SCL90R) is a general psychopathology self-assessment scale composed of 90 items, which investigates the presence of symptoms in the week before the test check. These 90 items, which have 5-level Likert answers, have 10 reference factors: (1) somatization (Som); (2) obsessive/compulsive (Obs); (3) interpersonal sensitivity (Interp Sens); (4) Depression (Dep); (5) anxious (Anx); (6) anger/hostility (Anger Host); (7) phobia (Phob); (8) psychoticism (Psych); (9) paranoia (Paran); and (10) sleep disorders. The score goes from 0 to 4, and a score above 1 is an index of pathology [32].

### 2.5. Composition of Probiotic Oral Suspension (POS)

The POSG received 3 g/day of probiotic oral suspension (POS) containing *Streptococcus thermophiles* (1.5 × 10^10^ colony-forming unit (CFU), CNCM strain number I-1630), *Bifidobacterium animalis subsp. Lactis* (1.5 × 10^10^ colony-forming unit (CFU)), *Bifidobacterium bifidum* (1.5 × 10^10^ colony-forming unit (CFU)), *Streptococcus thermophiles* (1.5 × 10^10^ colony-forming unit (CFU)), *Lactobacillus bulgaricus* (1.5 × 10^10^ colony-forming unit (CFU), CNCM strain numbers I-1632 and I-1519), *Lactococcus lactis subsp. Lactis* (1.5 × 10^10^ colony-forming unit (CFU), CNCM strain number I-1631), *Lactobacillus acidophilus* (1.5 × 10^10^ colony-forming unit (CFU)), *Lactobacillus plantarum* (1.5 × 10^10^ colony-forming unit (CFU)), *Lactobacillus reuteri* (1.5 × 10^10^ colony-forming unit (CFU), DSM 17938), corn maltodextrin, anticaking agent (silica), casein, lactose, and gluten < 3 ppm LLOQ (lower limit of quantitation) (Biocult Strong, HOMEOSYN, Rome, Italy).

The placebo was 3 g/day of inert material (flour type 00), maltodextrin from corn, anticaking agent (silica), casein, lactose, and gluten < 3 ppm LLOQ (lower limit of quantitation) (HOMEOSYN, Rome, Italy). The appearance of the placebo was indistinguishable in color, shape, size, packaging, smell, and taste from that of the probiotic supplement.

### 2.6. Statistical Analysis

Hardy-Weinberg equilibrium (HWE) was assessed using SNP-HWE program and tested using the *χ*^2^ analysis (Wigginton et al. 2005). To analyze the sample, subjects were divided into carriers (IL-1*β* rs16944, -598C) and noncarriers (IL-1*β* rs16944, -598T). The power of the study was calculated with the Quanto Program (USC Biostats, California, US). Shapiro-Wilk test was performed to determine parametric and nonparametric data. For comparisons between averages and medians, nonparametric tests for asymmetrically distributed data were conducted in all analyses and presented as mean (±standard deviation). In order to determine the presence of statistically significant differences among treatments and IL-1*β* carriers/noncarriers, *t*-test or Mann–Whitney test was performed. Percent frequency variation was analyzed using the McNemar and Pearson chi-square test. The association of IL-1*β* and the categorical Hamilton score was assessed by binary logistic regression (LBM) represented as odds ratio (OR) and 95% confidence intervals (CI). In all the statistical tests performed, the null hypothesis was rejected at the probability level greater than or equal to 0.05 (*p* ≥ 0.05). General Estimated Equations (GEE) were used to model the effects of risk and protective factor correlation between treatment, A carriers and noncarriers, time, and HAM-A results [33]. Statistical analyses were carried out using the IBM SPSS21.0 software for Windows (Armonk, NY: IBM Corp. USA).

## 3. Results

### 3.1. Population Characteristics

**Figure 1:**
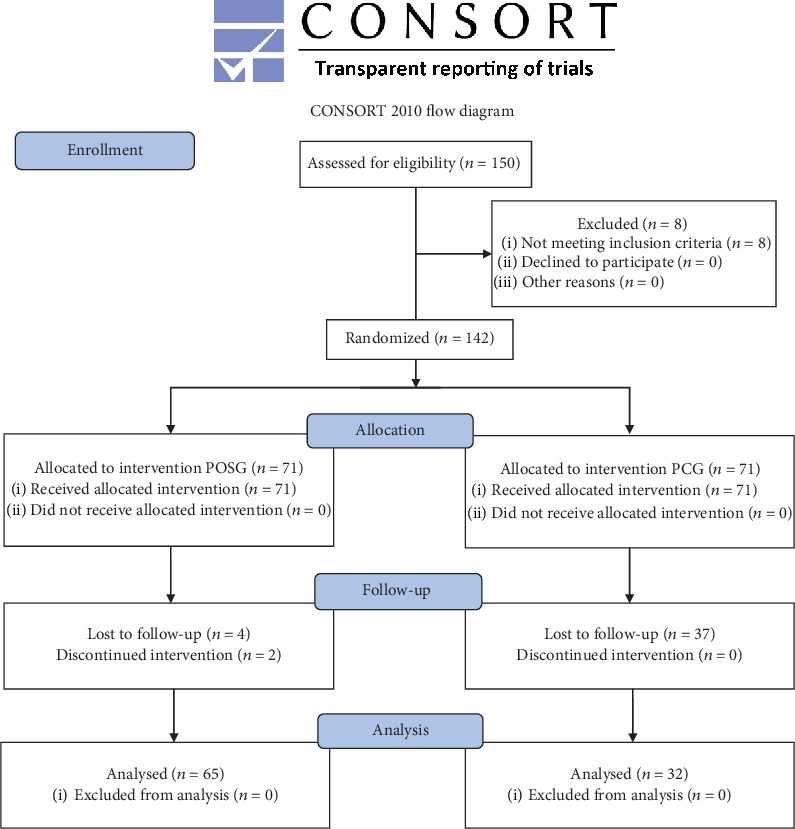
Study design. Consort flow diagram of the study. Probiotic oral suspension group (POSG) and placebo control group (PCG).

Out of the 150 patients recruited, 8 were excluded as they did not meet the inclusion criteria. The remaining 142 subjects were randomized equally into two groups. The first group (POSG) consumed the probiotic mixture formulation, and the second group (PCG) consumed the placebo formulation. During this clinical trial, 6 subjects from POSG and 34 subjects from PCG abandoned the study for the poor performances of treatments (Figure 1).

The final sample consisted of 97 patients, with ages ranging from 18 to 62 years old (POSG: mean 43.81 (±14.88), PCG: mean 32.92 (±11.75)). These patients successfully participated and completed the study protocol. At baseline, the total sample was divided according to A carrier and noncarrier for SNP rs16944. The tested SNPs of the IL-1*β* gene was in Hardy-Weinberg equilibrium (*p* > 0.05). The power of the study was 0.95, with fixed *α* = 0.05 and 2-sided. Genotype frequencies shown in TSI population (GG: 0.38, AA: 0.14, AG: 0.48) [34] are similar to the ones of our subjects (GG: 0.47, AA: 0.14, AG: 0.39), as well as the allele frequencies for TSI (A: 0.38; G: 0.62) and for our sample (A: 0.34; G: 0.66) (Table 1).

### 3.2. Influence of IL-1*β* Polymorphism on HAM-A, BUT, and SCL-90R Tests

The overall description of the total sample population at baseline can be seen in Table 2. Of the 65 subjects in POSG, 31 subjects were noncarrier (47.69%) and 34 (52.31%) A carrier. Of the 34 subjects in PCG, 15 (44.12%) subjects were noncarrier and 19 (55.88%) A carrier. At baseline, among treatment groups, no statistically significant difference (*p* ≥ 0.05) for total BUT score, BUT GSI score, total SCL-90R score, and SCL90R GSI score (Table 3) was highlighted. However, at baseline, there is a difference between frequencies of A carrier and non carrier for HAM-A within the two groups (*p* < 0.01) (Table 3). Moreover, A carriers, according to HAM-A, had significantly higher risk to be anxious compared to noncarriers (*p* < 0.01; OR = 5.90 (1.73; 20.16)) (Table 4), showing an interaction between IL-1*β* polymorphism and anxiety state. Frequencies of A carriers and noncarriers, according to psychometric results, before and after treatment, were reported in Table 5.

### 3.3. Effect of POS Treatment on HAM-A, BUT, and SCL-90R Questionnaires according to IL-1*β* SNP

After 12 weeks of intervention, we noticed an improvement in the psychometric parameters according to HAM-A test. POS treatment reduced score significantly (Table 3) and the frequency of anxious patients (Δ% = −10.64%), more than in PCG (Δ% = −5.10%) (Table 5).

**Figure 2:**
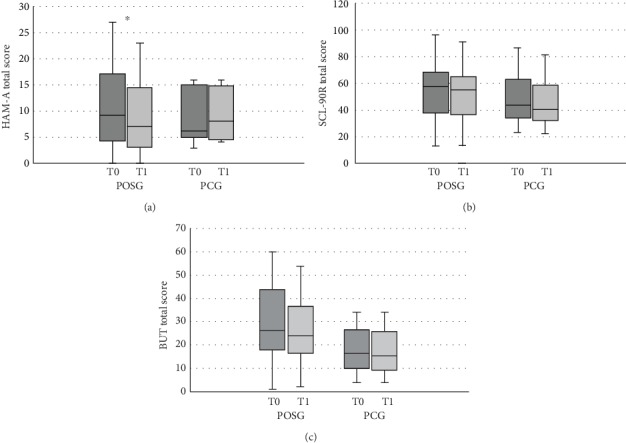
Comparison of psychometric test results at baseline and after treatment in POSG and PCG. Comparison of POSG and PCG before and after treatment. HAM-A: Hamilton Anxiety Rating Scale; SCL-90R: Symptom Checklist-90 Revised; BUT: Body Uneasiness Test; POSG: probiotic oral suspension group; PCG: placebo control group. Values are presented as median with min and max. Statistical significance attributed to results with ^∗^*p* < 0.05.

Furthermore, GEE analysis highlighted a significant reduction of the HAM-A total score after POS treatment compared to the PC (*β* = −0.33; *p* < 0.01; OR = 0.68 (0.40; 1.15)). POS treatment determined a significant reduction of anxiety risk in A carriers (*β* = −0.32; *p* = 0.02; OR = 0.73 (0.56; 0.94)), but not in noncarriers (*p* ≥ 0.05) (Table 6). These results highlighted the beneficial effect of POS treatment on anxiety state and the increased sensitivity of IL-1*β* A carriers to probiotic administration on anxiety reduction. Conversely, BUT and SCL-90 questionnaire results did not show significant changes after POS treatment compared to placebo (Table 3 and Figure 2).

## 4. Discussion

Among the most prevalent psychiatric disorders, anxiety is a condition that occurs worldwide, affecting the normal functioning of millions of people and burdening national health system economies. Despite the worldwide high prevalence, such disorders are often neglected and misdiagnosed. It is common for anxiety-affected individuals to be also suffering from other physical symptoms or concomitant mood disorders, like depressive conditions, drugs abuse, and even suicide (National Institute of Mental Health 2010).

Many studies focused on the role of inflammation on the CNS functions and relative diseases. In particular, IL-1*β* has pleiotropic effects on the CNS, where the proinflammatory cytokine, released by neurons and glial cells, acts in an autocrine and/or paracrine fashion, participates in the onset and progression of different neurodegenerative diseases and stroke [16]. Furthermore, proinflammatory cytokine expression and circulating levels, like interferon gamma (INF*γ*), TNF*α*, IL-6, and IL-1*β*, are associated with mood disorder symptoms [17].

In mouse models, IL-1*β* concentrations have been linked not only to neurodegenerative diseases but also to memory impairment [35] and anxiety disorders [36, 37].

In humans, IL-1*β* polymorphisms are linked to the levels of related cytokine expression and consequently to elevated risk of depression in different populations [25, 26, 28].

Nevertheless, the emerging knowledge of the MGB axis highlights the role of the gut microbiota as an important modulator of neuroinflammation, stress response, mood, and behavior and increases its importance in psychiatric disorder onset and progression, including anxiety [14, 15, 19, 21]. The gut microbiota is able to regulate systemic IL-1*β* concentrations [38], potentially modulating anxiety disorders [39–41].

Despite the numerous studies, the mechanism that links systemic inflammation and neurological disorders is still poorly understood, and nowadays, there is a gap in the scientific literature about the role of IL-1*β* in human anxiety.

In this study, we genotyped the IL-1*β* gene (rs16944) to observe the relationship of the polymorphism and anxiety state in an Italian population sample. At baseline, we found frequency differences between IL-1*β* A carriers and noncarriers, according to HAM-A scores (*p* < 0.01). In fact, anxious subjects were 43.24% A carriers and 11.43% noncarriers. At baseline, A carriers had moderate but significative increased risk to be anxious compared to noncarriers (5.90 (1.73; 20.16)).

Our observations implicate a bidirectional relationship between anxiety disorders and rs16944 polymorphism in an Italian population. The present results are in line with previous data shown by Kovacs et al. [29], which found a relationship between high life stress and anxiety symptoms, measured by the Brief Symptom Inventory, and the minor A allele of rs16944 polymorphism in Hungarian population. In that study, however, the increase in anxiety symptoms was related to childhood adversity, suggesting that both early life stress and the presence of the minor allele A are synergic contributing factors in disorder development. The number of studies investigating the role of IL-1*β* SNPs in anxiety disorders is few; hence, the present results should stimulate scientific interest on the influence of genetic asset and anxiety disorders.

In the light of this association, we investigated the combined effect of IL-1*β* polymorphism and probiotic administration in mood disorder phenotypes. Logan and Katzman assumed for the first time that the use of probiotics as adjuvant treatment in patients with major depressive disorder, a condition with complex pathophysiology associated with neurotransmitter and neuromodulator deficiencies, increased proinflammatory cytokine levels, gastrointestinal disturbances, and HPA axis dysfunction [42]. More recently, literature has shown that selective modulation of gut microbiota by exogenous agents, such as probiotic administration, could represent a novel therapeutic approach for mood and anxiety disorders [43, 44]. The beneficial effects of anxiety- and depression-related behavior are mainly obtained through the administration of the genera *Bifidobacterium* and *Lactobacillus*, but only some specific strains have ensured positive results [15]. Therefore, the secondary outcome of the present study was to evaluate the potential anxiolytic effect of the novel psychobiotic formulation to treat or prevent anxiety disorders, assessed with HAM-A scale, according to the IL-1*β* SNP rs16944.

After the 12-week intervention, we observed an improvement in the psychometric parameters. As determined by GEE analysis, HAM-A total score was significantly reduced in subjects who consumed the probiotic formulation (*p* < 0.01) compared to PCG results. Furthermore, the probiotic mixture lowered the percentage of anxious patients (Δ% = −10.64%), more than in PCG (Δ% = −5.10%) (Table 5).

These data suggest that probiotic intake has an impact on anxiety and confirmed our previous results of the concomitant administration of probiotics and hypocaloric diet in obese subjects [45] that suggested a greater improvement of anxiety symptoms.

For the evaluation of body image perception, BUT-A was performed both at baseline and on follow-up visit. As can be observed in Table 3, at baseline, BUT did not highlight a difference between POSG and PCG GSI score results (1.19 ± 1.02 and 0.52 ± 0.31, respectively), regardless of being A carrier or noncarrier. At the end of the 12-week intervention, we did not notice a significant reduction in BUT-A GSI in both POS and PC groups. Thus, we cannot conclude that the administered probiotic formulation is able to modify body image disorders. Moreover, our results are not in line with a previous study performed by De Lorenzo et al. [46], probably because the groups selected in that study included only women, making it difficult to compare the results, knowing the test's limitation according to Cuzzolaro et al. [31] in the male population. Therefore, in contrast to Messaoudi et al. [47], our GSI results of psychological distress measured by SCL-90R after 12-week intervention were not significant (*p* ≥ 0.05) in this study. In our opinion, those results can be explained by the low GSI score (GSI < 1) since the baseline time point (Table 3).

Family environment and genetics are established risk factors in the etiology of psychiatric disorders as well as anxiety development [48]. Multiple genes of small effect contribute to the disorder vulnerability, and the interaction between genetic and distressing environmental factors may lead to the onset of anxiety disorders [49]. There is a number of convincing studies that have recognized a direct transmission of anxiety within families, mainly observed in first-degree relatives, with an overall four- to sixfold increased risk [50]. The genetic contribution to the pathophysiology of psychiatric disorders is highly complex. Previous studies found higher risk of depression in IL-1*β* gene rs16944 carriers of the higher synthesizing A allele, in schizophrenia [25] and Alzheimer disease patients [26].

Interestingly, in this study, after the 12-week probiotic intake, IL-1*β* A carriers, but not noncarriers, had a significant reduction of HAM-A score (*p* = 0.02), and the frequency percentage of anxious carriers has been cut from 37.93% to 21.74% (Table 5). Although we cannot exclude an independent impact of the minor A allele of rs16944 on microbiota composition and modulation, our results suggest that the psychobiotic administration determined a reduction of anxiety and related symptoms and restored psychological equilibrium in the treated sample.

In conclusion, despite the limitations related to the lack of IL-1*β* blood level measurement in this clinical study, our results suggest that the consumption of probiotics mitigates anxiety symptoms, especially in healthy adults with the minor A allele of rs16944 as a risk factor. This study provides further evidences that gut microbiota is involved in the psychological state and that its modulation may improve the overall quality of life. Furthermore, the 12-week intervention was sufficient to afford significant results without manifestation of adverse events, and so, the psychobiotic intake represents a good approach to attenuate anxiety-related feelings. Thus, probiotics might serve as a new therapeutic approach for neuropsychiatric disorder treatment and/or prevention. Although preclinical data suggest the benefits of probiotic use in anxiety-related disorders, clinical evidence is somewhat lacking as well as the establishment of which probiotic strains clearly have psychobiotic properties. In the light of these observations, clinical studies on the role of psychobiotics in anxiety are at the very least necessary in order to establish more accurately the probiotic therapeutic efficiency.

The research field related to gut microbiota manipulation and mood disorders is far from exhausted. Hence, our results are aimed at further contributing to the scientific evidences on psychobiotic ability to manage anxiety disorders and improve related symptomatology and identifying the potential mechanisms implicated. The next step would be the assessment of the minor A allele of rs16944 on microbiota composition and modulation and then the “psychobiotics” effect of probiotics compared to anxiolytic drugs on anxiety-diagnosed subjects, to further confirm their psychotropic properties.

## Figures and Tables

**Table 1:** Study population allele and genotype frequencies for IL-1*β* rs16944 compared to Tuscan Italians from Southern Europe (TSI).

IL-1*β* rs16944		
Allele frequency	A	G
TSI	0.38	0.62
Study population	0.34	0.66
Genotype frequency	AA	AG
TSI	0.14	0.48
Study population	0.14	0.39

**Table 2:** Descriptive characteristics of recruited study population.

Parameter (*n* = 97)	Mean (±SD)
Gender (%)	Female = 61.9%
	Male = 38.1%
Age	41.29 (±14.90)
Total BUT score	37.17 (±33.36)
BUT GSI score	1.09 (±0.98)
Total SCL-90R score	61.83 (±47.33)
SCL-90R GSI score	0.69 (±0.53)
Hamilton score	10.91 (±7.31)

Descriptive table. Results are expressed in mean ± SD. BUT: Body Uneasiness Test (BUT); SCL90R : Symptom Checklist-Revised; HAM-A: Hamilton Anxiety Rating Scale.

**(a):** 

	POSG	PCG		POSG			PCG			A carrier POSG vs. PCG	Noncarrier POSG vs. PCG
	Baseline	Baseline		A carrier	Noncarrier		A carrier	Noncarrier			
			*p*	Baseline	Baseline	*p*	Baseline	Baseline	*p*	*p*	*p*
Total BUT score	40.42 ± 13.81	37.67 ± 10.67	0.133	42.16 ± 41.46	40.65 ± 22.89	0.871	41.67 ± 37.64	49.67 ± 25.86	0.721	0.605	0.231
BUT GSI score	1.19 ± 1.02	0.52 ± 0.31	0.123	1.45 ± 1.22	0.90 ± 0.67	0.091	0.76 ± 1.22	0.78 ± 0.17	0.925	0.128	0.085
Total SCL-90R score	64.17 ± 50.19	47.83 ± 21.54	0.441	78.53 ± 58.81	68.12 ± 43.18	0.325	62.67 ± 40.60	63.30 ± 38.72	0.374	0.325	0.525
SCL-90R GSI score	0.71 ± 0.56	0.53 ± 0.24	0.146	0.87 ± 0.65	0.53 ± 0.37	0.565	0.70 ± 0.23	0.67 ± 0.10	0.567	0.095	0.525
Hamilton score	10.77 ± 7.63	11.50 ± 5.91	0.738	13.03 ± 8.00	8.50 ± 6.63	0.025	14.38 ± 4.37	7.67 ± 5.75	0.012	0.401	0.217
Age	43.81 ± 14.88	37.92 ± 11.75	0.091								

**(b):** 

	POSG						PCG					
	A carrier			Noncarrier			A carrier			Noncarrier		
	Baseline	T1	*p*	Baseline	T1	*p*	Baseline	T1	*p*	Baseline	T1	*p*
Total BUT score	42.16 ± 41.46	47.42 ± 43.11	0.787	40.65 ± 22.89	35.83 ± 40.40	0.565	41.67 ± 37.64	45.09 ± 45.89	0.521	49.67 ± 25.86	43.33 ± 3.51	0.258
BUT GSI score	1.45 ± 1.22	1.39 ± 1.27	0.341	0.90 ± 0.67	0.91 ± 1.19	0.566	0.76 ± 1.22	1.33 ± 1.35	0.128	0.78 ± 0.17	0.83 ± 0.10	0.162
Total SCL-90R score	78.53 ± 58.81	74.36 ± 58.22	0.140	68.12 ± 43.18	59.60 ± 62.56	0.374	62.67 ± 40.60	63.54 ± 58.76	0.296	63.30 ± 38.72	60.33 ± 42.52	0.624
SCL-90R GSI score	0.87 ± 0.65	0.83 ± 0.65	0.302	0.53 ± 0.37	0.64 ± 0.69	0.064	0.70 ± 0.23	0.82 ± 0.65	0.652	0.67 ± 0.10	0.90 ± 0.33	0.136
Hamilton score	13.03 ± 8.00	9.33 ± 7.42	0.001	8.50 ± 6.63	6.07 ± 6.61	0.152	14.38 ± 4.37	14.13 ± 4.26	0.351	7.67 ± 5.75	7.33 ± 5.28	0.363

Results are expressed in mean ± standard deviation. *t*-test was used for all parameters. Significant values are for *p* < 0.05. POSG: probiotic oral suspension group; PCG: placebo control group; BUT: Body Uneasiness Test; SCL90R: Symptom Checklist-Revised; HAM-A: Hamilton Anxiety Rating Scale; BUT GSI: Body Uneasiness Test Global Severity Index; SCL90R GSI: Symptom Checklist-Revised Global Severity Index.

**Table 4:** IL-1*β* A carrier risk for depression, dysmorphic, and anxiety symptoms.

	*χ* ^2^ value	*χ* ^2^ *p*	*β*	SE	*p*	OR (minimum-maximum)	*R* ^2^
BUT	1.91	0.17	0.92	0.67	0.17	2.50 (0.67; 9.31)	0.06
SCL-90R	1.63	0.20	0.97	0.78	0.21	2.64 (0.58 12.09)	0.06
HAM-A	9.08	<0.01	1.78	0.63	<0.01	5.90 (1.73; 20.16)	0.18

IL-1*β* A carrier risk for depression, dysmorphic, and anxiety symptoms evaluated with Body Uneasiness Test (BUT), Symptom Checklist-Revised (SCL90R), and Hamilton Anxiety Rating Scale (HAM-A).

**Table 5:** BUT, SLC-90R, and HAM-A frequencies.

			BUT		SCL-90		HAM-A	
			Healthy	Dysmorphic symptoms	Healthy	Depressive symptoms	Healthy	Anxiety symptoms
A carrier	Total population	T0	54.55%	45.45%	68.18%	31.82%	56.76%	43.24%
		T1	61.54%	38.46%	66.67%	33.33%	70.97%	29.03%
		*Δ*%	-6.99%		1.52%		-14.21%	
	PCG	T0	100.00%	0.00%	100.00%	0.00%	37.50%	62.50%
		T1	100.00%	0.00%	100.00%	0.00%	50.00%	50.00%
		*Δ*%	0.00%		0.00%		-12.50%	
	POSG	T0	47.37%	52.63%	63.16%	36.84%	62.07%	37.93%
		T1	58.33%	41.67%	63.64%	36.36%	78.26%	21.74%
		*Δ*%	-10.96%		-0.48%		-16.19%	

Noncarrier	Total population	T0	75.00%	25.00%	85.00%	15.00%	88.57%	11.43%
		T1	88.89%	11.11%	87.50%	12.50%	93.10%	6.90%
		*Δ*%	-13.89%		-2.50%		-4.53%	
	PCG	T0	100.00%	0.00%	100.00%	0.00%	100.00%	0.00%
		T1	100.00%	0.00%	100.00%	0.00%	100.00%	0.00%
		*Δ*%	0.00%		0.00%		0.00%	
	POSG	T0	70.59%	29.41%	82.35%	17.65%	86.21%	13.79%
		T1	83.33%	16.67%	80.00%	20.00%	91.30%	8.70%
		*Δ*%	-12.75%		2.35%		-5.10%	

Total	Total population	T0	64.29%	35.71%	76.19%	23.81%	72.22%	27.78%
		T1	64.29%	35.71%	76.19%	23.81%	81.67%	18.33%
		*Δ*%	0.00%		0.00%		-9.44%	
	PCG	T0	100.00%	0.00%	100.00%	0.00%	64.29%	35.71%
		T1	100.00%	0.00%	100.00%	0.00%	71.43%	28.57%
		*Δ*%	0.00%		0.00%		-7.14%	
	POSG	T0	58.33%	41.67%	72.22%	27.78%	74.14%	25.86%
		T1	66.67%	33.33%	68.75%	31.25%	84.78%	15.22%
		*Δ*%	-8.33%		3.47%		-10.64%	

Frequencies for positive/negative classification on depression, dysmorphic, and anxiety symptoms evaluated with Body Uneasiness Test (BUT), Symptom Checklist-Revised (SCL90R), and Hamilton Anxiety Rating Scale (HAM-A). Results are expressed as percentage. POSG: psychobiotic oral suspension group; PCG: placebo control group.

**Table 6:** Association of IL-1*β* and POSG treatment with HAM-A results.

	*β*	Error SD	*p*	OR (minimum-maximum)
POSG vs. PCG	-0.33	0.11	<0.01^∗^	0.68 (0.40; 1.15)
POSG vs. PCG in IL-1*β* rs16944 noncarriers	-0.29	0.19	0.12	0.75 (0.52; 1.08)
POSG vs. PCG in IL-1*β* rs16944 A carriers	-0.32	0.13	0.02^∗^	0.73 (0.56; 0.94)

HAM-A results associated with polymorphism rs16944 within the IL-1*β* gene with 12 weeks in the psychobiotic oral suspension group (POSG). GEE analysis for HAM-A results, significant values (^∗^*p* ≤ 0.05) are expressed for POSG vs. placebo control group (PCG), POSG vs. PCG in IL-1*β* rs16944 noncarrier group, and POSG vs. PCG in IL-1*β* rs16944 carrier group.

## Data Availability

The data used to support the findings of this study are included within the article.

## Abbreviations

ANS: Autonomic nervous system
BUT: Body Uneasiness Test
CFU: Colony-forming unit
CNS: Central nervous system
DNA: Deoxyribonucleic acid
ENS: Enteric nervous system
GAD: Generalized anxiety disorder
GI: Gastrointestinal
HAM-A: Hamilton Anxiety Rating Scale
HPA: Hypothalamic-pituitary-adrenal
IL: Interleukin
LPS: Lipopolysaccharide
MGB: Microbiota-gut-brain
POS: Probiotic oral suspension
SCL: Symptom Checklist
SNP: Single-nucleotide polymorphism.
